# Risk and predictors of severe hyperkalemia after total parathyroidectomy without auto-transplantation in patients with secondary hyperparathyroidism

**DOI:** 10.3389/fendo.2024.1463735

**Published:** 2024-10-18

**Authors:** Chenchen He, Longfei Li, Junhao Pan, Guangming Cheng, Chunhui Wang, Yufu Tang

**Affiliations:** ^1^ Department of Clinical Medicine and Surgery, China Medical University, Shenyang, China; ^2^ Department of Hepatobiliary and Thyroid Surgery, General Hospital of Northern Theater Command, Shenyang, China

**Keywords:** secondary, hyperparathyroidism, parathyroidectomy, hyperkalemia, risk factors

## Abstract

**Objective:**

To identify the risk factors of postoperative severe hyperkalemia after total parathyroidectomy (TPTX) without auto-transplantation in patients with secondary hyperparathyroidism (SHPT).

**Methods:**

Data on 406 consecutive patients who underwent TPTX without auto-transplantation for secondary hyperparathyroidism at the General Hospital of Northern Theater Command between January 2013 and January 2023, were prospectively collected. Then, patients were divided into the training set (n=203) and the validation set (n=203) in a ratio of 1:1 by timeline. The patients were divided into severe hyperkalemia group and non-hyperkalemia group according to the postoperative serum kalium level >6.0 mmol/L with ECG changes or serum kalium level ≥6.5 mmol/L. Univariate and multivariate logistic regression analyses were used to evaluate the possible risk factors associated with postoperative severe hyperkalemia after TPTX. The predictive performance was evaluated with receiver operating characteristic (ROC) curves with the areas under the ROC curve (AUC) and calibration curve. Decision curve and clinical impact curve analyses were used to validate the clinical application of the value.

**Results:**

The incidence of postoperative severe hyperkalemia was 15.5% in all patients, 17.2% and 13.8% in the training and validation cohorts, respectively. The risk factors associated with postoperative severe hyperkalemia was higher preoperative kalium level. The optimal cut-off value for preoperative serum kalium level was 5.0mmol/L according to the ROC curve. The area under the curve (AUC) achieved good concordance indexes of 0.845 (95%CI, 0.776-0.914) in the training cohort. The sensitivities were 0.829 (95%CI: 0.663-0.934) and 0.857 (95%CI: 0.673-0.960) in the training and validation cohorts, respectively. The specificities were 0.798 (95%CI: 0.729-0.856) and 0.720 (95%CI:0.647-0.785) in the training and validation cohorts, respectively. Calibration curve exhibited a good consistency between actual observations and predicted severe hyperkalemia in the training and validation cohorts.

**Conclusions:**

Our study found that the preoperative kalium levels is only a risk factor for postoperative severe hyperkalemia in patients undergoing TPTX for secondary hyperparathyroidism. The threshold for preoperative serum kalium levels is 5.0mmol/L that can serve as a useful indicator for identifying patients with severe hyperkalemia after surgery. These results provide valuable suggestion for clinical practice.

## Introduction

Parathyroidectomy is an effective treatment for secondary hyperparathyroidism when patients cannot tolerate drug therapy or develop drug resistance (1, 2). Hyperkalemia is one of the most serious complications after parathyroidectomy (PTX). It has an incidence of 20%-50% and is associated with paresthesia, muscle weakness, suffocation, arrhythmia and even leading to cardiac arrest (3–6).

In the last decade, it has been reported that age, gender, preoperative blood kalium and blood calcium levels and stage of renal failure can help predict the postoperative hyperkalemia after parathyroidectomy with autotransplantation (PTX + AT) (7–9). Some studies have reported that preoperative blood kalium is a risk factor after PTX and Perioperative values could not predict postoperative hyperkalemia with the need for urgent hemodialysis (UHD) (6, 10). Moreover, the criteria for defining hyperkalemia vary in each study, and the thresholds for predicting outcome variables also different. In addition, hyperkalemia is classified into three levels: mild, moderate, and severe (11). Severe hyperkalemia is more worthy of attention. Thus, due to the lack of high level of evidence and unified standards, the risk factors for severe hyperkalemia are still unclear and controversial.

Here, we report a single center regression study. The main purpose of this study is to explore the risk factors for severe hyperkalemia after TPTX+AT based on a large amount of case data from our center. The optimal threshold for risk factors would be determined, providing a theoretical basis for clinical practice.

## Materials and methods

### Patients

Between January 2013 and January 2023, data on consecutive patients with SHPT who had undergone TPTX in the General Hospital of Northern Theater Command were collected. The study was approved by the Institutional Ethics Committee of the General Hospital of Northern Theater Command (No.:Y(2022)197). All patients were informed of the risks and procedures of the surgery and signed informed consent.

The inclusion criteria were as follows: (1) in accordance with the Kidney Disease Outcomes Quality Initiative guidelines, patients with persistently elevated serum intact PTH (iPTH) levels >800 pg/ml, uncontrolled hypercalcemia with hyperphosphatemia, severe clinical symptoms such as bone and joint pain, muscle weakness, or refractory pruritus, or refractory to medical treatment, need to undergo PTX; (2) the surgery is technically successful with the iPTH value of <60  pg/mL on postoperative day 1 (POD1). (3) Patients who underwent TPTX with auto-transplantation. Patients who underwent second PTX due to recurrent SHPT following the initial PTX, underwent a failure operation and had incomplete clinical data were excluded. Successful TPTX is defined as a number of resected parathyroid glands ≥ 3, and an iPTH value of < 60 pg/mL 24 h post-surgery (9).

### Clinical variables

We collected preoperative information on clinical variables, including gender, age, body mass index (BMI), dialysis duration, and preoperative laboratory tests (serum intact parathyroid hormone, serum alkaline phosphatase, serum calcium, serum phosphate, serum kalium, hemoglobin, albumin, serum creatinine, urea, prothrombin and fibrinogen). All patients had dialysis within 24 h before surgery. Severe hyperkalemia was defined as serum kalium level >6.0 mmol/L with ECG changes or serum kalium level ≥6.5 mmol/L. Levels of serum kalium, serum calcium, and serum phosphorous were measured for three days in the morning after surgery. If postoperative hyperkalemia was diagnosed, patients would be treated according to the management of KDIGO (11).

### Surgical procedures

Routine preoperative examination included hemoglobin, albumin, prothrombin, fibrinogen, serum intact PTH level, concentrations of calcium, blood phosphorus, and renal function tests, ultrasonography of the thyroid and parathyroid glands. The preoperative diagnosis was based on criteria of the KDIGO clinical practice guideline (1, 12). All surgical procedures were performed by Dr. Guangming Cheng and his surgical team.

### Statistical analysis

Continuous variables were expressed as means and standard deviations or medians with interquartile ranges (IQR) as appropriate. Categorical variables were summarized as the counts and percentages in each category. Continuous variables were compared by Student’s t test or Mann-Whitney U test, whereas categorical variables were compared by chi-squared test or Fisher’s exact test, as appropriate. Univariable analysis was used to identify clinically relevant variables associated with postoperative hyperkalemia in the training cohort. All variables associated with hyperkalemia at a significant level were candidates for multivariate logistic analysis. In addition, ROC curve analysis was performed to obtain the AUC and calibration with 1000 bootstrap samples to decrease the overfit bias. The optimal cutoff value was defined according to the analysis of ROC curve. The clinical application value of this model was validated using decision curve and clinical impact curve analyses. All statistical analyses were performed by using R software (version 4.3.1), and a *P* value of less than 0.05 was considered to be statistically significant.

## Results

### Patient’s characteristics

In total, 406 patients were enrolled in this study. The clinical characteristics of all patients in **Table 1**. Men and women comprise 57.6% and 42.4% with a median age of 46.0 (37.0-53.5) years old in the training cohort. The validation cohort consists of 51.2% males and 48.8% females with a median age of 49.0 (38.5-56.0) years old. The postoperative severe hyperkalemia rates were 17.2% and 13.8% in the training and validation cohorts, respectively. The clinical characteristics of the patients in the two cohorts are listed in **Table 2**.

**Table 1:** Clinical characteristics of 406 patients undergoing TPTX.

Variable	All patients (n=406)
Gender, %	
Male	221 (54.4)
Female	185 (45.6)
Initial kidney disease, %	
Nephritis	111 (27.3)
Hypertensive nephropathy	40 (9.9)
Diabetic nephropathy	17 (4.2)
Polycystic kidney	18 (4.4)
Others	28 (6.9)
Unknow	192 (47.3)
Age,median (IQR), y	47.0 (37.0-55.8)
BMI,median (IQR), kg/m^2^	22.1 (19.6-24.8)
Duration of dialysis, median (IQR), y	8.00 (6.00-10.0)
Hemoglobin, mean (SD), g/L	106 (17.4)
Alkaline phosphatase, median (IQR), U/L	277 (164-543)
Albumin, median (IQR), g/L	38.4 (35.7-40.8)
Blood urea nitrogen, median (IQR), mmol/L	20.9 (16.7-26.0)
Serum creatinine, median (IQR), μmol/L	926 (756-1119)
Prothrombin time, median (IQR), s	13.4 (12.9-13.9)
Fibrinogen, median (IQR), g/L	4.37 (3.72-5.00)
iPTH, median (IQR), pg/ml	1864 (1402-1900)
Serum calcium, median (IQR), mmol/L	2.46 (2.31-2.58)
Serum kalium, median (IQR), mmol/L	4.67 (4.18-5.12)
Serum phosphate, median (IQR), mmol/L	2.34 (2.03-2.80)
Severe hyperkalemia, %	
No	343 (84.5)
Yes	63 (15.5)

BMI, body mass index; iPTH, intact parathyroid hormone; IQR, interquartile range; SD, standard deviation.

**Table 2:** Clinical characteristics of patients in training and validation cohorts undergoing TPTX.

Variable	Cohort, No. (%)		*P* value
	Training	Validation	
	N=203	N=203	
Gender			0.232
Male	117 (57.6)	104 (51.2)	
Female	86 (42.4)	99 (48.8)	
Age,median (IQR), y	46.0 (37.0-53.5)	49.0 (38.5-56.0)	0.107
BMI,median (IQR), kg/m^2^	22.1 (19.9-24.8)	22.0 (19.4-24.8)	0.578
Duration of dialysis, median (IQR), y	8.00 (6.00-10.0)	8.00 (6.00-10.0)	0.921
Hemoglobin, mean (SD), g/L	107 (18.0)	106 (16.9)	0.423
Alkaline phosphatase, median (IQR), U/L	261 (155-554)	296 (175-538)	0.336
Albumin, median (IQR), g/L	38.7 (35.6-41.2)	38.0 (35.7-40.5)	0.460
Blood urea nitrogen, median (IQR), mmol/L	21.1 (16.9-26.2)	20.6 (16.4-25.8)	0.482
Serum creatinine, median (IQR), μmol/L	937 (771-1148)	897 (735-1096)	0.072
Prothrombin time, median (IQR), s	13.5 (12.9-14.0)	13.4 (12.8-13.9)	0.204
Fibrinogen, median (IQR), g/L	4.27 (3.60-5.02)	4.41 (3.88-4.97)	0.283
iPTH, median (IQR), pg/ml	1797 (1391-1900)	1900 (1418-1900)	0.325
Serum calcium, median (IQR), mmol/L	2.46 (2.34-2.57)	2.46 (2.30-2.58)	0.786
Serum kalium, median (IQR), mmol/L	4.73 (4.19-5.09)	4.62 (4.17-5.12)	0.536
Serum phosphate, median (IQR), mmol/L	2.34 (1.99-2.80)	2.35 (2.08-2.79)	0.449
Severe hyperkalemia			0.411
No	168 (82.8)	175 (86.2)	
Yes	35 (17.2)	28 (13.8)	

BMI, body mass index- iPTH, intact parathyroid hormone- IQR, interquartile range- SD, standard deviation.

### Prediction of postoperative hyperkalemia

All variables used in this analysis were based on the data obtained preoperatively. The univariate analyses revealed that albumin (OR=1.114, 95%CI: 1.013-1.234, P=0.032), blood urea nitrogen (OR= 1.089, 95%CI: 1.030-1.556, P=0.003), Prothrombin time (OR= 0.643, 95%CI: 0.417-0.975, P=0.040) and preoperative Serum kalium (OR= 8.281, 95%CI: 4.139-18.728, P<0.001) were significantly associated with postoperative severe hyperkalemia (**Table 3**). All of the above-mentioned significant parameters were then put into multivariate logistic regression analysis. The results showed that preoperative serum kalium (OR= 7.066, 95% CI: 3.438−16.361, P<0.001) was independent risk factors associated with postoperative severe hyperkalemia (**Table 4**).

**Table 3:** Univariate logistic regression analysis in the training cohort.

Variable	OR (95%CI)	*P* value
Gender	0.443(0.748-3.256)	0.235
Age, median (IQR), y	0.993(0.961-1.025)	0.660
BMI, median (IQR), kg/m2	0.945(0.851-1.041)	0.270
Duration of dialysis, median (IQR), y	1.020(0.913-1.135)	0.724
Hemoglobin, mean (SD), g/L	1.021(0.999-1.043)	0.056
Alkaline phosphatase, median (IQR), U/L	1.000(0.999-1.001)	0.960
Albumin, median (IQR), g/L	1.114(1.013-1.234)	0.032
Blood urea nitrogen, median (IQR), mmol/L	1.089(1.030-1.556)	0.003
Serum creatinine, median (IQR), μmol/L	1.000(0.999-1.002)	0.328
Prothrombin time, median (IQR), s	0.643(0.417-0.975)	0.040
Fibrinogen, median (IQR), g/L	1.040(0.734-1.445)	0.821
iPTH, median (IQR), pg/ml	1.000(0.999-1.001)	0.739
Serum calcium, median (IQR), mmol/L	0.390(0.060-2.589)	0.324
Serum kalium, median (IQR), mmol/L	8.281(4.139-18.728)	<0.001
Serum phosphate, median (IQR), mmol/L	1.318(0.708-2.433)	0.378

iPTH, intact parathyroid hormone; OR, odds ratio; CI, confidence interval.

**Table 4:** Multivariate logistic regression analysis in the training cohort.

Variable	β	OR (95%CI)	P value
Albumin, g/L	0.113	1.120(1.001−1.265)	0.056
Blood urea nitrogen, mmol/L	0.051	1.053(0.987−1.126)	0.120
Prothrombin time, s	-0.357	0.700(0.402−1.211)	0.203
Serum kalium, mmol/L	1.955	7.066(3.438−16.361)	<0.001

OR, odds ratio; CI, confidence interval.

### ROC and calibration curve analysis

**Figure 1:**
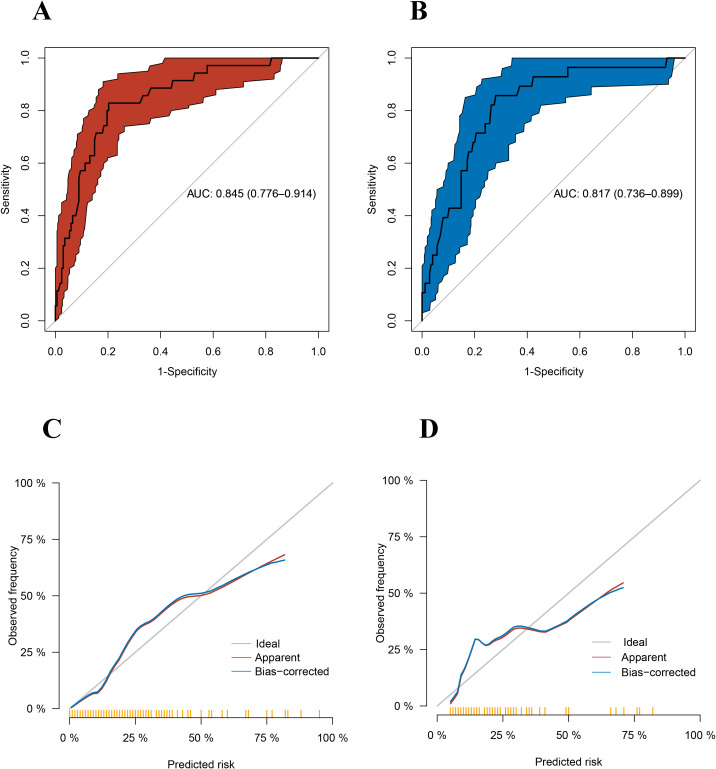
**(A)** Receiver operating characteristics (ROC) of the model in the training cohort. **(B)** Receiver operating characteristics (ROC) of the model in the validation cohort. **(C)** The calibration curve of the model in the training cohort. **(D)** The calibration curve of the model in the validation cohort.

ROC curve analysis revealed that, at an optimal cutoff value for preoperative kalium of 5.0mmol/L. In the training cohort, there were 63 patients with preoperative serum kalium levels ≥ 5.0mmol/L, 29 patients with severe hyperkalemia after surgery, 140 patients with preoperative serum kalium levels<5.0 mmol/L, and 6 patients with severe hyperkalemia after surgery. In the validation cohort, there were 64 patients with preoperative serum kalium levels ≥ 5 mmol/L, 21 patients with severe hyperkalemia after surgery, 139 patients with preoperative serum kalium levels<5 mmol/L, and 7 patients with severe hyperkalemia after surgery. The AUC achieved good concordance indexes of 0.845 (95%CI, 0.776-0.914) (**Figure 1A**) and 0.817% (95%CI: 0.736-0.899) (**Figure 1B**) in the training and validation cohort, respectively. The sensitivities were 0.829 (95%CI: 0.663-0.934) and 0.857 (95%CI: 0.673-0.960) in the training and validation cohorts, respectively. The specificities were 0.798 (95%CI: 0.729-0.856) and 0.720 (95%CI:0.647-0.785) in the training and validation cohorts, respectively. (**Table 5**). Calibration curve exhibited a good consistency between actual observations and predicted severe hyperkalemia in the training and validation cohorts (**Figures 1C, D**).

**Table 5:** Accuracy of the prediction for the risk of severe hyperkalemia presence.

Variable	Value (95%CI)	
	Training Cohort	Validation Cohort
Area under ROC curve	0.845(0.776-0.914)	0.817(0.736-0.899)
Sensitivity, %	0.829(0.663-0.934)	0.857(0.673-0.960)
Specificity, %	0.798(0.729-0.856)	0.720(0.647-0.785)
Positive predictive value, %	0.460(0.368-0.715)	0.329(0.259-0.660)
Negative predictive value, %	0.957(0.901-0.971)	0.970(0.915-0.978)
Positive likelihood ratio	4.094(2.926-5.728)	3.061(2.310-4.057)
Negative likelihood ratio	0.215(0.103-0.447)	0.918(0.080-0.494)

### DCA and CIC curve analysis

**Figure 2:**
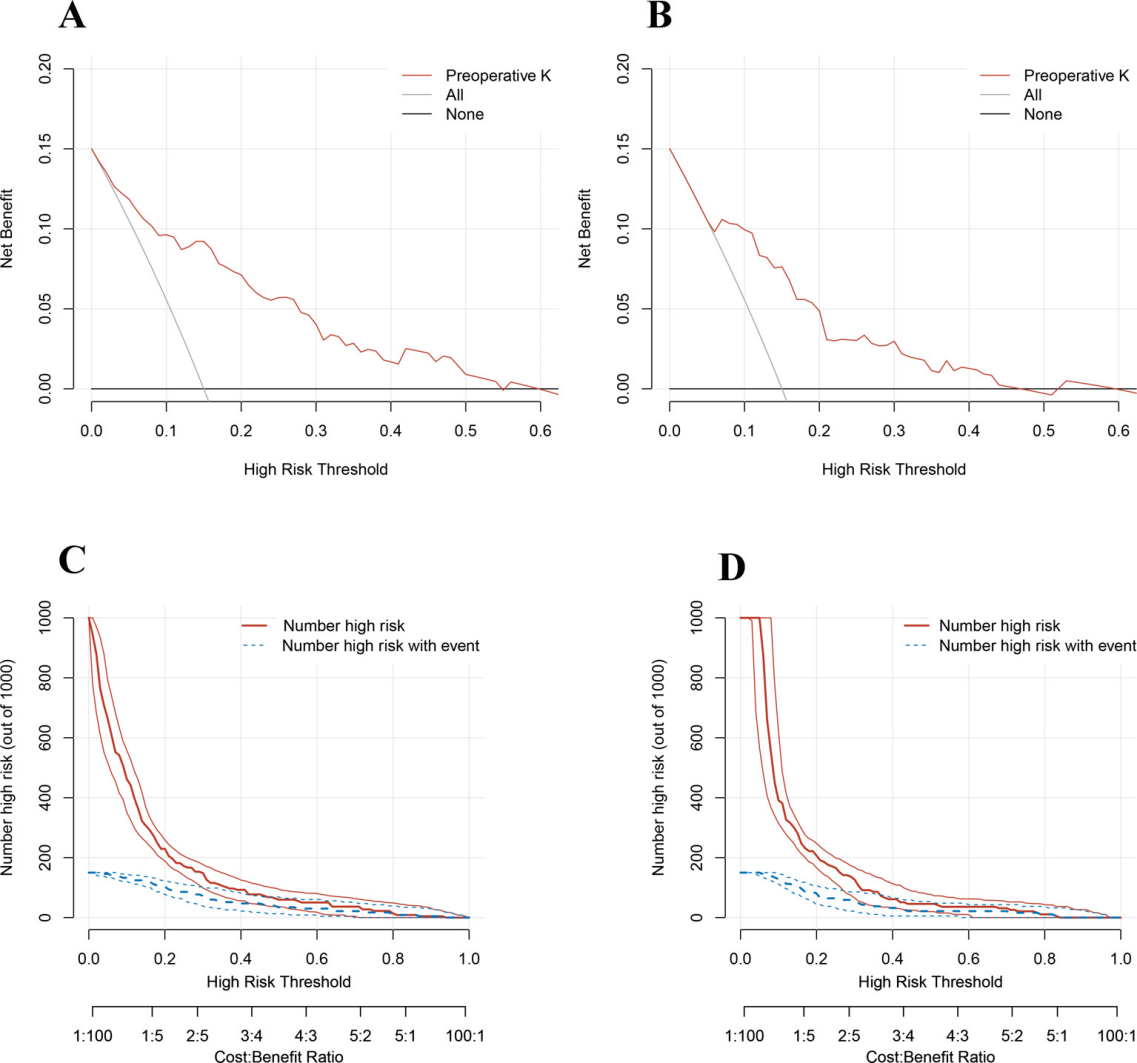
**(A)** Decision curve analysis of the model in the training cohort. **(B)** Decision curve analysis of the model in the validation cohort. **(C)** Clinical impact curve of the model in the training cohort. **(D)** Clinical impact curve of the model in the validation cohort.

Decision curve analysis (DCA) and clinical impact curve (CIC) were used to validate the clinical application value of the model. When the threshold value in the range of 5%-55%, the model had a good net benefit (**Figures 2A, B**). As shown in **Figures 2C, D**, the model also showed greater clinical net benefits, which further demonstrated that the model had better predictive and accuracy values.

## Discussion

In this retrospective study, the incidence of severe hyperkalemia in our study is 15.5%, only preoperative high levels of kalium are the risk factors for severe hyperkalemia after TPTX without auto-transplantation. The optimal cutoff value for preoperative blood kalium is 5.0mmol/L. To reduce the incidence of severe postoperative hyperkalemia, we recommend adjusting the dialysis frequency prior to surgery, performing routine dialysis on the day before operation, and avoiding the intake of high-kalium foods. These measures are intended to ensure that serum kalium levels are maintained below 5.0mmol/L.

The incidence of hyperkalemia in CKD patients is 15-20% (13). If treated properly, patients with mild transient hyperkalemia have a good prognosis. Sudden onset of extreme hyperkalemia can lead to muscle weakness, paralysis, or arrhythmia. If not treated quickly, it can be fatal in up to two-thirds of cases. Hyperkalemia is an independent risk factor for death in hospitalized patients (14).

Previous studies have explored the risk factors for post PTX hyperkalemia, but most studies define hyperkalemia based on the upper limit of normal values. In CKD patients, patients with higher frequency of hyperkalemia may have better adaptation mechanisms to combat its harmful effects (15). Therefore, compared to mild hyperkalemia, it is more important to focus on severe hyperkalemia.

In the past decade, the risk factors for hyperkalemia after PTX remained in question. Hayes et al. suggested that all patients undergoing parathyroidectomy should be carefully observed for possible hyperkalemia. However, they did not delve into the specific risk factors (16). Bajaj et al. conducted that the comparison between the kalium measurement after anesthesia induction and the first intraoperative kalium measurement was a good predictive factor for postoperative kalium increase and the need for glucose-insulin therapy (17). This suggests that high kalium may be a risk factor for postoperative hyperkalemia. Yang et al. reported that younger age and male gender were risk factors for intraoperative hyperkalemia (5). Li et al. observed that preoperative high kalium and age were risk factors for postoperative hyperkalemia. The AUC value for preoperative high kalium was 0.783, with a sensitivity of 77.8% and a specificity of 71.1%. Age, as a risk factor, had an AUC value of only 0.375 in single-factor analysis and thus could not be considered an independent risk factor (3). Song et al. found that preoperative high kalium was an independent risk factor for postoperative hyperkalemia. The ROC curve revealed an optimal value of 3.9 mmol/L with good sensitivity and specificity (6). Zhu et al. analyzed that dialysis time, preoperative kalium level, and blood calcium level were associated with postoperative hyperkalemia (7). It is worth noting that the definition of hyperkalemia in all studies mentioned above were different. Most guidelines define hyperkalemia as kalium levels greater than 5.0mmol/L now (11, 18–22). The definition of hyperkalemia using the upper limit of normal kalium levels overlooks the potential risk, especially for patients with renal insufficiency or end-stage renal disease who have impaired kalium regulation. Therefore, the use of threshold values for defining hyperkalemia is necessary.

Severe hyperkalemia is absolutely the most dangerous in complications after PTX. Previous studies only focus on the happen of hyperkalemia or not but ignore the severity. This study aims to solve this problem. To avoid relative heterogeneity, we strictly follow the guidelines to screen the included patients. Patients were divided into training and validation cohorts according to the timeline. The result showed that preoperative high levels of blood kalium are the only risk factor for severe hyperkalemia after surgery. The AUC value for postoperative severe hyperkalemia was 0.845, with a sensitivity of 82.9% and a specificity of 79.8%. The optimum cut off value is 5.0mmol/L. It preformed also well in the validation cohort.

There are many reasons leading to high level of preoperative kalium. Firstly, in acute kidney injury and chronic kidney failure, patients are prone to elevated blood kalium levels due to decreased glomerular filtration rate or dysfunction of renal tubular kalium excretion. Renal tubular diseases can also cause a relative deficiency of adrenal mineralocorticoids, a low response to aldosterone, and kalium excretion disorders in the renal distal tubules and collecting ducts, leading to an increase in blood kalium levels (23). Secondly, CKD is often accompanied by metabolic acidosis. At this time, the patient’s extracellular hydrogen ion concentration is high, which promotes the transfer of intracellular kalium ions to the extracellular space to maintain charge balance, thereby causing an increase in blood kalium levels (24). Thirdly, food intake is the main source of kalium ions. High kalium intake may lead to an increase in blood kalium when renal excretion is impaired. Several common drugs are known to cause or aggravate hyperkalemia such as Renin-angiotensin-aldosterone system inhibitors (RAASIs), nonsteroidal anti ⁃ inflammatory drugs (NSAIDs) and the like (25, 26). Therefore, in order to reduce the occurrence of severe postoperative hyperkalemia, it is particularly important to manage blood kalium levels before surgery.

In our study, we conducted postoperative blood kalium measurements on the first day after surgery. Theoretically, changes in blood kalium levels after surgery can occur within a few hours. Therefore, we recommend monitoring changes in blood kalium six hours or even earlier after surgery. For patients whose preoperative blood kalium levels did not drop below 5.0mmol/L, monitoring of postoperative blood kalium changes as soon as possible is recommended. Once it exceeds 5.5mmol/L, glucose combined with insulin therapy can be used. For patients with postoperative blood kalium levels above 6.5mmol/L, dialysis combined with medication such as sodium zirconium silicate should be performed for treatment.

Our study had some limitations. First, this analysis was based on data from a single institution; it is necessary to validate the results from other centers. Second, only a risk factor was identified. The high level of kalium before surgery is the result of a combination of various factors such as concomitant therapy, dietary preferences and the like, which need to be further explored. Third, the vast majority of patients included in our study were end-stage patients, it is unclear whether different stages of renal failure have the same effect on postoperative severe hyperkalemia.

## Conclusions

Our study found that the preoperative kalium levels is only a risk factor for postoperative severe hyperkalemia in patients undergoing TPTX for secondary hyperparathyroidism. The threshold for preoperative serum kalium levels is 5.0mmol/L that can serve as a useful indicator for identifying patients with severe hyperkalemia after surgery. These results provide valuable suggestion for clinical practice.

## Data Availability

The raw data supporting the conclusions of this article will be made available by the authors, without undue reservation.
